# Secondary neurons are arrested in an immature state by formation of epithelial vesicles during neurogenesis of the spider *Cupiennius salei*

**DOI:** 10.1186/1742-9994-1-3

**Published:** 2004-10-25

**Authors:** Angelika Stollewerk

**Affiliations:** 1 Abteilung fuer Evolutionsgenetik, Institut fuer Genetik, Universitaet zu Koeln, Weyertal 121, 50931 Koeln, Germany; 2 Department of Zoology, University of Cambridge, Downing Street, Cambridge, CB2 3EJ, uk

**Keywords:** neural precursors, invagination, epithelial vesicles, glial cells, chelicerate, *Cupiennius salei*

## Abstract

**Background:**

In the spider *Cupiennius salei *about 30 groups of neural precursors are generated per hemi-segment during early neurogenesis. Analysis of the ventral neuromeres after invagination of the primary neural precursor groups revealed that secondary neural precursors arise during late embryogenesis that partially do not differentiate until larval stages.

**Results:**

In contrast to the primary groups, the secondary invaginating cells do not detach from each other after invagination but maintain their epithelial character and form so-called epithelial vesicles. As revealed by dye labeling, secondary neural precursors within epithelial vesicles do not show any morphological features of differentiation indicating that the formation of epithelial vesicles after invagination leads to a delay in the differentiation of the corresponding neural precursors. About half of the secondary neural precursor groups do not dissociate from each other during embryogenesis indicating that they provide neural precursors for larval and adult stages.

**Conclusions:**

Secondary neural precursors are arrested in an immature state by formation of epithelial vesicles. This mechanism facilitates the production of larval neural precursors during embryogenesis. I discuss the evolutionary changes that have occured during neural precursor formation in the arthropod group and present a model for the basal mode of neurogenesis.

## Background

The arthropods form a diverse group with a correspondingly high variation of neural structures adapted to the specialized behaviour and lifestyles of individual species. This raises the question of how developmental processes have been modified during evolution to generate the wide diversity of nervous systems seen in adult arthropods. Evolutionary modifications that lead to variations in neural structures can occur during different processes of neurogenesis. The establishment of neural networks can be influenced by changes in the generation of neural precursors, modifications of cell fates or elimination of individual neurons as well as changes in axonal guidance. A comparative analysis of neurogenesis in chelicerates and myriapods has revealed that although the developmental program is genetically conserved, there is a major difference in the recruitment of neural precursors as compared to insects and crustaceans [1-5]. Groups of neural precursors invaginate from the ventral neuroectoderm in a regular, strikingly similar pattern in spiders (chelicerates) and myriapods, while in insects and crustaceans single neural precursors are selected. This modification may be the basis for variations in the functions of spider and myriapod neurons, since a comparison of early segmentally repeated neurons that pioneer the major axon tracts in crustaceans and insects has not revealed any similarities in cell body positions or axonal outgrowths to myriapod neurons [6,7].

In the spider 30 to 32 groups of neural precursors are generated per hemi-segment during neurogenesis. As in *Drosophila melanogaster*, the neural precursors arise at stereotyped positions that are prefigured by a proneural gene (*CsASH 1*), while the neurogenic genes *Delta *and *Notch *restrict the proportion of cells that adopt the neural fate at each wave of neural precursor formation [1,2]. In *Drosophila melanogaster*, the Delta/Notch signalling pathway is used for a decision between two cell fates in the ventral neuroectoderm: delaminating cells become neural precursors, while cells that remain apical give rise to epidermis. This decision does not take place in the central neurogenic regions of the spider [2]. The epidermal cells are derived from lateral regions that overgrow the neuromeres after invagination of the neural precursors.

Since each invagination group consists of five to nine neural precursors, it can be estimated that an embryonic hemineuromere consists of about 220 neurons on average, similar to *Drosophila*. However, in the adult spider *Cupiennius salei *the subesophageal ganglion consists of 49,000 neurons [8] indicating that over 40,000 neurons must be generated during late embryonic and larval stages. In *Drosophila melanogaster*, 'embryonic' neuroblasts proliferate again and give rise to larval and adult lineages after a phase of cell cycle arrest from late embryogenesis to first larval instar [9-11]. An analysis of the mitotic pattern during neurogenesis has revealed that neuroblasts are missing in the spider [1]. In addition, most of the neural precursors do not divide after invagination. This raises the question of how additional neurons are generated that contribute to the larval and adult CNS of the spider.

## Results

In the spider *Cupiennius salei *the germband develops from aggregations of cells that form the cephalic lobe and the caudal lobe [12]. One to three prosomal segments are generated by a subdivision of the cephalic lobe, while the remaining segments arise sequentially from the caudal lobe, the so-called posterior growth zone [12,13]. At the beginning of neurogenesis (about 130 hours of development; stages after Seitz [12]) a longitudinal furrow forms that divides the germband into left and right parts that remain connected only at the cephalic lobe and the posterior growth zone. The two halves of the embryo move laterally until they finally meet at the dorsal midline (ca. 300 hours of developement). This process is called inversion [12]. The formation of neural precursors and the invagination of these cells occurs during inversion [1].

### Secondary invagination sites form after invagination of the primary neural precursors

**Figure 1:**
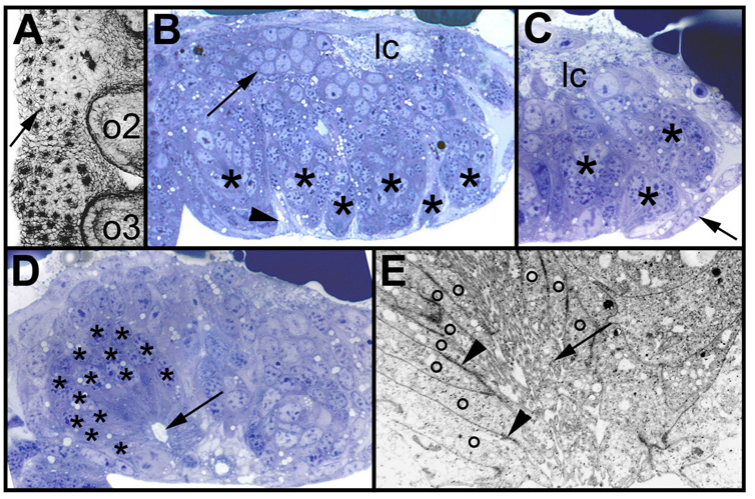
(A-E): Morphology of the secondary invagination sites. Confocal micrograph (A, inverted) of a flat preparation of an embryo stained with phalloidin-rhodamine and light micrographs (B-D) and electron micrograph (E) of transverse sections through prosomal hemi-neuromeres. The midline is to the right. (A) Final pattern of the primary invagination sites in the opisthosomal segments 1 and 2. The invagination sites are arranged in 7 rows. The black dots correspond to the constricted cell processes of the individual precursor groups that are attached to the apical surface (arrow). (B) Morphology of the secondary invagination sites. At 250 hours the secondary invaginating cell groups (asterisks) are still attached to the apical surface. The individual groups are isolated by brighter sheath cells (arrowhead). The primary precursor groups have dissociated (arrow) and form basal cell layers. The longitudinal connective (lc) is already visible at the basal side. (C) The secondary invagination sites (asterisks) loose contact to the apical surface, when the epidermis (arrow) overgrows the ventral neuromeres. (D) After invagination the secondary neural precursors (asterisks) remain attached to each other forming epithelial vesicles. The cell processes run parallel to each other and extend to a lumen (arrow). (E) The cell processes (o) of the invaginating cells of a group are opposed to each other and the lumen between the cell processes is filled with microvilli (arrow). Cell junctions connect the individual processes (arrowheads). *lc*, longitudinal connective; *o2 *to *o3*, opisthosomal hemi-segments 2 to 3.

Although the invagination sites in the ventral neuroectoderm of the spider are generated in four subsequent waves over a time period of three days, the neural precursors detach form the apical surface at about the same time between 200 and 230 hours of development. Fig. 1A shows the final arrangement of the invagination sites shortly before the neural precursors loose contact to the apical surface. After invagination, the neural precursors differentiate and a neuropil develops at the basal side of the neuromeres (Fig. 1B, arrow). Since the epidermis arises lateral and medial to the ventral hemi-neuromeres (Fig. 1C, arrow), all cells of the central neurogenic region are eventually incoporated into the ganglion, i.e., the ventral neuroectoderm does not give rise to epidermoblasts and neuroblasts as in *Drosophila melanogaster *(see above). A detailed analysis of the morphology of the ventral neuromeres after invagination of the neural precursor groups revealed that the cells that remain apical form secondary invagination sites (Fig. 1B, asterisks).

**Figure 2:**
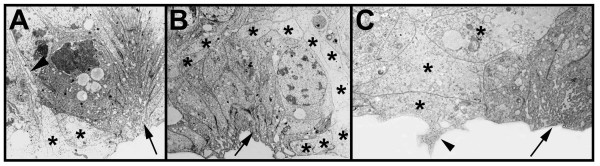
(A-C): Secondary invagination sites are surrounded by sheath cells. Electron micrographs of transverse sections through prosomal hemi-neuromeres. (A,B) Invagination sites (arrows) are surrounded by sheath cells (asterisks) that appear translucent in the electron microscope. The sheath cells extend processes (arrowhead) that enwrap the individual invagination sites. (C) Sheath cells that are located in the apical cell layer form bizarre shapes that extend into the cell free space at the ventral side of the embryo (arrowhead). The sheath cells are labeled with asterisks, the arrow points to an invagination site.

**Figure 3:**
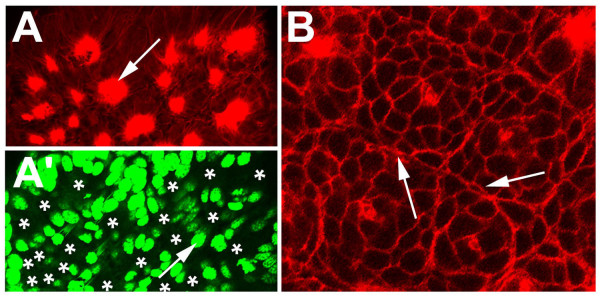
(A-B): The nuclei of cells within the secondary invagination sites are located basally. Confocal micrographs of flat preparations of embryos double-stained with phalloidin-rhodamine (red) and YOYO (green) (A,A') and single stained with phalloidin-rhodamine (B). (A,A') The apical optical section at 250 hours of development shows that the secondary invagination sites (arrow) are still attached to the apical surface. The nuclei of the secondary precursors are located basally, as revealed by the absence of nuclei staining in the apical cell layer. The asterisks in A' indicate the positions of the cell processes of the secondary invagination sites (compare to A). (B) The basal optical section shows the distinct morphology of the sheath cells (arrows) that subdivide the individual invagination sites.

**Figure 4:**
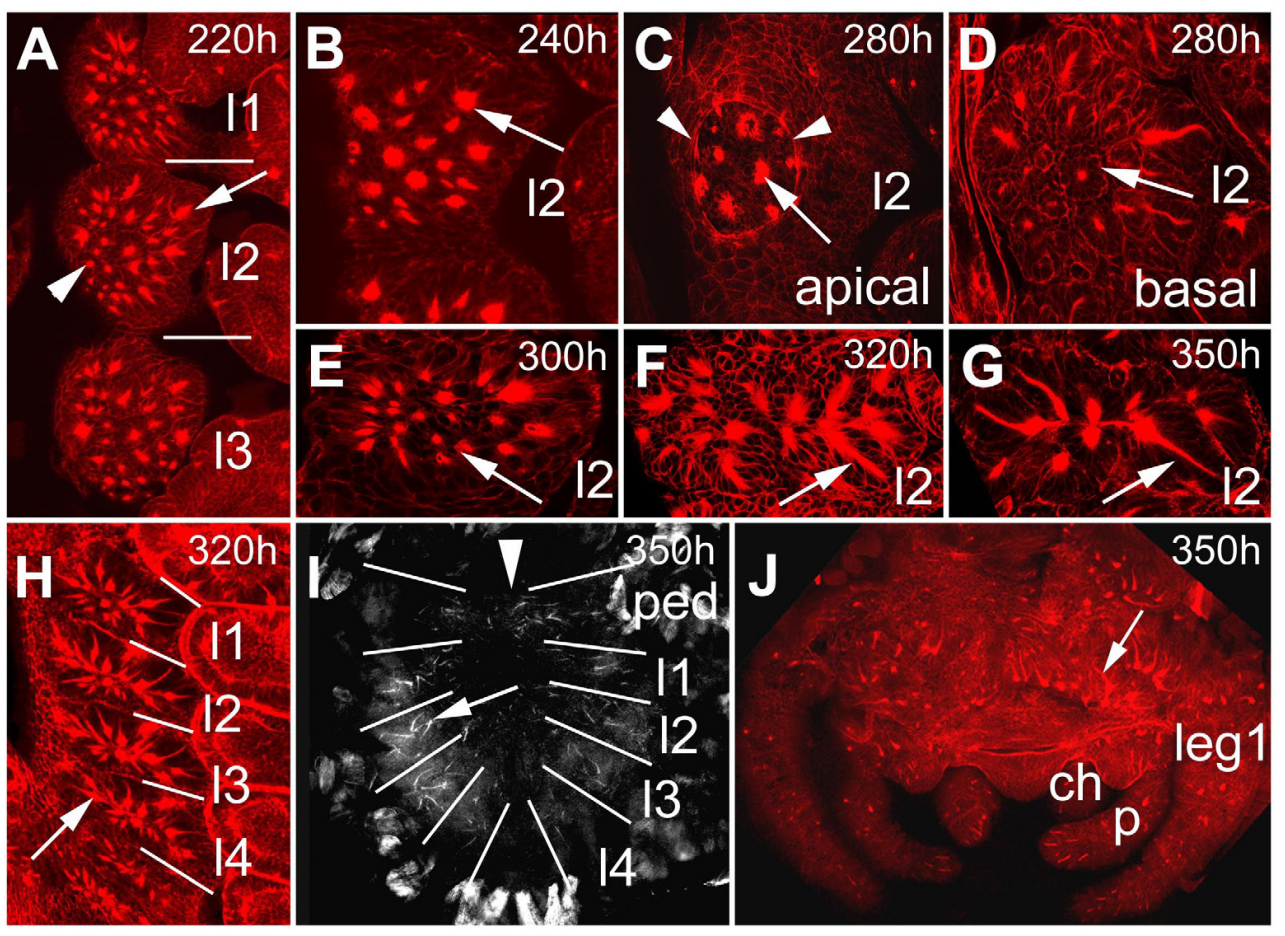
(A-j): Invagination of secondary neural precursors and formation of epithelial vesicles. (A-F) Confocal micrographs of flat preparations of embryos stained with phalloidin-rhodamine. (B-G) Flat preparations of the fourth prosomal hemi-segments. (A) At 220 hours about 25 secondary invagination sites form (arrow). There is no clear dividing line between the formation of secondary invagination sites (arrow) and invagination of primary neural precursors. Some primary invagination sites are still visible (arrowhead) The bars indicate the segment borders. (B) Apical optical section of the pattern of secondary invagination sites (arrow) at 240 hours of development. (C) Epidermal cells overgrow the ventral neuromeres between 250 and 300 hours (arrowheads) The arrow points to a secondary invagination group. (D) After invagination the individual cells of a groups remain attached to each other forming epithelial vesicles (arrow). (E) At 300 hours the anterior-posterior extension of the individual hemi-segments has been reduced leading to a rearrangement in the positions of the invaginated cell groups (arrow). (F) After 320 hours 8 of the 25 invaginated cell groups are no longer visible indicating that the cells have detached from each other. The arrow points to an invaginated cell group. (G) 10 cell groups are still visible at hatching (arrow). (H) Overview of the arrangement of epithelial vesicles (arrow) of the four prosomal hemi-segments corresponding to the four walking legs. The anterior-posterior reduction in size is clearly visible (compare to A). The bars indicate the segment borders. (I) Flat preparation of the prosoma at hatching. Epithelial vesicles are still visible (arrow). The bars indicate the segment borders, the arrowhead points to the midline. (J) Flat preparation of the brain at 350 hours. The arrow points to epithelial vesicles. *ch*, chelicera; *l1 *to *l4*, prosomal neuromeres corresponding to walking leg 1 to 4; *leg 1*, walking leg 1.*p*, pedipalp; *ped*, pedipalpal neuromere.

The secondary invagination sites can be distinguished from the primary invagination groups by several morphological features. (1) Each secondary invagination group contains up to 40 cells as compared to 5 to 8 cells that form the primary invagination sites (Stollewerk et al., 2001; Fig. 1B,1D; see also Fig. 4F). (2) The cell processes of the secondary neural precursors do not extend straight to the apical surface as the primary invaginating cells, but face each other (Fig. 1D,1E). Microvilli extend into the lumen between the opposite cell processes (Fig. 1E, arrow). (3) While the primary neural precursors detach from each other after invagination, the secondary invaginating cell groups remain attached to each other and maintain their epithelial character. (Fig. 1D,1E) The individual cell processes are connected by cell junctions (Fig. 1E, arrow heads). (4) In contrast to the primary precursors, the secondary invaginating cell groups are surrounded by sheath cells. These cells are visible in the light and electron microscope as brighter cells that separate the individual invagination sites (Fig. 1B, arrowhead; Fig. 2, asterisks; Fig. 3B). They extend long cell processes that ensheath each cell group (Fig. 2A, arrowhead). Interestingly, the sheath cells that are located in the apical cell layer form bizarre cytoplasmic shapes that extend into the cell-free space at the ventral side of the embryo (Fig. 2C, arrow). Double-stainings with a marker for cell nuclei and a dye that stains the actin cytoskeleton show that the nuclei of the secondary neural precursors are shifted basally similar to the primary invagination sites (Fig. 3A,A'; see also Fig. 1B,1D). The stained nuclei that surround the individual secondary invagination sites in the apical cell layer correspond to the sheath cells (Fig. 3A,A').

### Secondary invagination sites persist as epithelial vesicles

**Figure 5:**
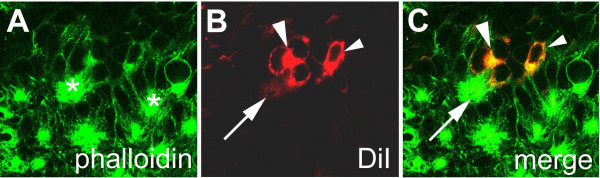
(A-C): DiI-labeling of cells within epithelial vesicles. Flat preparation of the fourth prosomal hemi-neuromere of an embryo labeled with DiI (red) and stained with phalloidin-rhodamine (green). (A-C) Invaginated cells in 40 segments of 10 embryos were labelled with DiI (red) and stained with phalloidin-FITC (green). The cells of a group (A, asterisks) are attached to each other (B,C large arrow head) and their short, thin cell processes run parallel to each other (B,C arrow). They do not show any morphological features of differentiation, i.e. they do not grow long thin dendritic or axonal processes. The small arrows (B,C) point to a cell of an adjacent invagination group.

In contrast to the primary invaginating cell groups that are generated in four waves (see above), the secondary invagination sites appear almost at the same time (Fig. 4A). A detailed analysis of the ventral neuromeres of embryos stained with phalloidin-rhodamine, a dye that stains the actin cytoskeleton and accumulates in the constricted cell processes of the invaginating cells, revealed, that about 25 invagination sites are generated per hemi-segment. There is no clear dividing line between the formation of secondary invagination sites and invagination of the primary neural precurors. At 220 hours of development all secondary invagination sites are visible (compare Fig. 4A and 4B), while some of the primary precursor groups are still attached to the apical surface (Fig. 4A, arrowhead). However, at 240 hours all primary precursors have detached from the apical surface and dissociated (Fig. 4B; see also Fig. 1B). At about 250 hours, epidermal cells arise lateral and medial to the ventral neuromeres and overgrow the ventral nerve cord within 50 hours [2]. In Fig. 4C (280 hours of development) the border of the overgrowing epidermis is visible as a circle in the medial region of each hemi-neuromere. Although the secondary invaginating cell groups detach from the apical surface at this time, the individual cells of a group remain attached to each other and persist as epithelial vesicles (Fig. 4D; see also Fig. 1C). Due to morphogenetic movements at about 300 hours of development, the anterior-posterior extension of the individual hemi-segments is reduced leading to a rearrangement of the position of the epithelial vesicles (Fig. 4E,4F,4G,4H). After 350 hours 8 of the 25 invaginated cell groups are no longer visible indicating that the cells have detached from each other (Fig. 4F,4H). However, 10 cell groups are still visible at hatching (Fig. 4G,4I). Similar to the ventral neuromeres, groups of cells invaginate from the cephalic lobe neuroectoderm and persist as epithelial vesicles until larval stages (Fig. 4J). DiI labelling of cells within epithelial vesicles revealed that the cells of a group are attached to each other (Fig. 5A,5B,5C) and their short, thin cell processes run parallel to each other (Fig. 5B, arrow). They do not show any morphological features of differentiation, i.e. they do not grow long thin dendritic or axonal processes. These data show that 10 groups of neural precursors per hemi-segment do not differentiate during embryogenesis but give rise to neural cells that will be incorporated into the larval ganglia.

### Dissociation of epithelial vesicles is not associated with cell divisions

**Figure 6:**
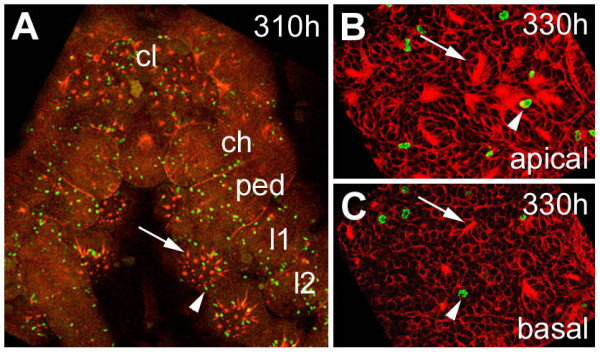
(A-C): Mitotic pattern in the ventral neuromeres after formation of the secondary invagination sites. Flat preparations of embryos stained with phalloidin-rhodamine (red) and anti-Phospho-Histon 3 (green). (A) Only scattered mitotic cells (arrowhead) are present in the ventral neuromeres after invagination of the secondary neural precursors (arrow). The pattern of cell divisions in the cephalic lobe and the prosomal segments at 310 hours of development is representative for the late embryonic stages. (B) Optical section through apical cell layers of the fourth prosomal hemi-neuromere. Only a few mitotic cells (arrowhead) are associated with epithelial vesicles. (C) A similar pattern is visible in basal cell layers of the same neuromere. The arrowhead points to a dividing cell, the arrow points to a dissociating epithelial vesicle. *ch*, cheliceral neuromere; *cl*, cephalic lobe; *l1 *to *l2*, prosomal hemi-neuromeres corresponding to walking legs 1 to 2; *ped*, pedipalpal hemineuromere.

Analysis of the mitotic pattern during neurogenesis has revealed that the formation of the primary invagination sites is not connected with cell divisions [1]. In addition, mitotic activity seems to be restricted to the apical layer of the ventral neuroectoderm with the exception of a few cells indiciating that most of the invaginated primary neural precursors differentiate without further divisions. However, there are two waves of mitosis during the course of neurogenesis [1]. After formation of most of the primary invagination sites many cell divisions can be observed in groups of cells and single cells in the apical cell layer. The second wave arises when the primary precursors detach from the apical surface and the secondary invagination sites begin to form. Cell divisions are also restricted to the apical cell layer with the exception of a few cells [1]. These data indicate that the number of neuroectodermal cells is increased by cell proliferation prior to the recruitment of secondary neural precursors. A further analysis of the mitotic pattern during late embryogenesis with the mitotic marker anti-Phospho-Histon 3 and phalloidin-rhodamine revealed that only scattered mitotic cells are present in the ventral neuromeres. The pattern of cell divisions in the cephalic lobe and the prosomal segments (310 hours of development) shown in Fig. 6A is representative for the late embryonic stages. Since only a few mitotic cells are associated with dissociating epithelial vesicles (Fig. 6B,6C), it can be assumed that the secondary precursors differentiate without further divisions, similar to the primary neural precursors.

### *achaete-scute *homologues and neurogenic genes are re-expressed during formation of the secondary precursors

Two *achaete-scute *homologues have been identified in the spider [1]. *CsASH1 *is expressed like a proneural gene in the neurogenic regions prior to formation of the primary invagination sites and is necessary for the generation of neural precursors. *CsASH2*, in contrast, shows a pan-neural mode of expression: it is exclusively expressed in all invaginating neural precursors. Simlar to *Drosophila melanogaster*, the neurogenic genes *Notch *and *Delta *restrict the proportion of cells that adopt a neural fate at each wave of neural precursor formation [2].

**Figure 7:**
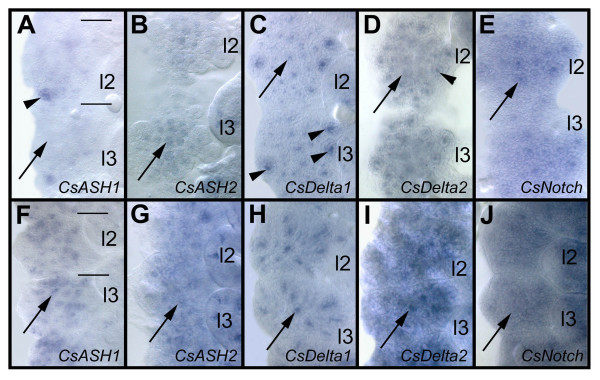
(A-J): Proneural and neurogenic genes are re-expressed during formation of the secondary neural precursors. Flat preparations of the fourth and fifth prosomal hemi-segments after in situ hybridisation of whole embroys. (A-E) 220 hours of development, (F-J) 250 hours of development. Anterior is at the top, the midline to the left. (A) At 220 hours, *CsASH1 *expression has been down-regulated in all primary neural precursors (arrow) with the exception of one group (arrowhead). (B) At this time the pan-neural gene *CsASH2 *is still weakly expressed in the primary neural precursors (arrow). (C) *CsDelta *transcripts accumulate in the secondary invagination sites (arrow heads), while transcripts are down-regulated in the primary precursor groups. (D) A similar expression, although weaker, is visible after *CsDelta2 *in situ hybridisation. The arrow points to a region where *CsDelta2 *has been down-regulated, the arrowhead indicates expression in the secondary neural precursors. (E) *CsNotch *remains expressed at low levels in the ventral neuroectoderm. An up-regulation of *CsNotch *transcripts is visible in the secondary invagination groups (arrow). (F) At 250 hours *CsASH1 *expression can be detected in the secondary invagination sites (arrow), although it is not expressed in all of them. (G) *CsASH2 *seems to be expressed weakly in all secondary invaginating cells groups (arrow). (H) A high accumulation of *CsDelta1 *transcripts is visible in about 10 of the invagination sites (arrow), (I) while *CsDelta2 *seems to be xpressed in all invagination groups (arrow). (J) *Cs Notc*h transcripts can be detected in all neuroectodermal cells at this time. *l2 *to *l3*, walking leg 2 to 3.

During formation of the secondary invagination sites, the spider *achaete-scute *homologues and neurogenic genes [1,2] are re-expressed in the ventral neuromeres (Fig. 7). After invagination of the primary neural precursors, the expression of the *achaete-scute *homologues *CsASH1 *and *CsASH2 *and the neurogenic genes *CsDelta1 *and *CsDelta2 *is down-regulated, while *CsNotch *remains expressed at low levels in the ventral neuroectoderm (Fig. 7A,7B,7C,7D,7E). There is no clear dividing line between the invagination of the primary neural precursors and the formation of the secondary invagination sites (see above), which is also obvious by *CsDelta1 *staining: while *CsDelta1 *is down-regulated in the primary neural precursors (Fig. 7C, arrow), transcripts accumulate at high levels in the secondary invagination sites (Fig. 7C, arrowheads). Similarily, a transient stronger expression of *CsNotch *is visible in the secondary invaginating cell groups (Fig. 7E, arrow). Interestingly, *CsASH1 *is only expressed after formation of the secondary invagination sites, in single cells and groups of cells (Fig. 7F, arrow) while the gene shows a proneural mode of expression during formation of the primary precursors [1]. Like *CsASH1*, *CsASH2 *shows a pan-neural expression in the invaginating secondary precursors (Fig. 7G, arrow). *CsDelta1 *transcripts accumulate only in a subset of the secondary invaginating cell groups while *CsDelta2 *seems to be expressed in all of them (Fig. 7H,7I). *CsNotch *shows a ubiquituous expression in the ventral neuromeres (Fig. 7J).

## Discussion

### Formation of epithelial vesicles – a conserved character in arthropod neurogenesis?

Analysis of the ventral neuromeres of spider embryos after invagination of the primary neural precursor groups revealed that secondary neural precursors arise during late embryogenesis that partially do not differentiate until larval stages. In contrast to the primary groups, the secondary invaginating cells do not detach from each other after invagination but maintain their epithelial character. In common with epithelial cells, they show a pronounced apico-basal polarity. The apical surface is covered with microvilli, while the lateral surfaces adhere to those of neighbouring cells of a group via specialized cell junctions, i.e. zonulae adhaerentes.

Although the formation of epithelial cell groups has not been observed in the ventral neuromeres of other arthropods, epithelial vesicles have been described during development of the stomatogastric nervous system and the brain in *Drosophila melanogaster*. After invagination of the individual neuroblasts that pioneer the frontal connective and recurrent nerve [14], three groups of cells invaginate from the stomatogastric nervous system primordium [15]. They loose contact with the surrounding stomodeal epithelium and form elongated, hollow epithelial vesicles, similar to the secondary neural precursors of the spider. Finally, they dissociate into apolar cells and are incorporated into different stomatogastric ganglia [15,16]. In a similar way, the vesicle forming the optic lobe invaginates from the posterior head region of *Drosophila melanogaster *embryos. In contrast to the stomatogastric vesicles, this cell group remains epithelial throughout embryogenesis and larval life [17].

It has been shown in *Drosophila melanogaster *that the Delta-Notch signaling pathway is involved in maintaining the epithelial character of the optic lobe and stomatogastric nervous system (SNS) precursors [16]. In *Notch *mutant *Drosophila melanogaster *embryos, cells with the identity of SNS and optic lobe precursors develop at approximately normal numbers, but they do not form epithelial vesicles. Instead, these cells appear as solid, irregular clusters of apolar cells [15-17]. In the spider, the function of *CsNotch *during development of the secondary neural precursors could not be analysed, because injection of ds *CsNotch *RNA leads to a premature differentiation of neural precursors due to an ealier function of *CsNotch *in lateral inhibition [2]. However, the up-regulation of *CsNotch *in the secondary invagination sites suggests a role in formation of the epithelial vesicles (see Fig. 7E).

Similar to *Notch*, the proneural genes *achaete*, *scute *and *lethal of scute *are continuously expressed in the SNS of *Drosophila melanogaster *[18]. Loss of proneural gene function leads to the absence of a subpopulation of SNS precursors and subsequently to an irregular invagination of the SNS placode. Furthermore, proneural genes seem to promote the dissociation of SNS precursors from the epithelial vesicles, since loss of proneural gene function results in a delay of this process. Similar to *Drosophila melanogaster*, both *achaete-scute *homologues of the spider are expressed in the epithelial vesicles that are formed by the secondary neural precursors. However, in contrast to its function in the recruitment of the primary neural precurors, the expression pattern of the spider proneural gene *CsASH1 *does not suggest a role in the establishment of the secondary neural fate. *CsASH1 *transcripts can only be detected in subsets of neural precursors after generation of the secondary invagination sites. A similar expression pattern can be observed for *CsASH2*, although the transcripts in the primary neural precursors are down-regulated later than the *CsASH1 *transcripts. The function of these two genes during generation of the secondary invagination sites and the formation of the epithelial vesicles could not be analysed. Due to their ealier role in the recruitment and differentiation of the primary precurors, injection of ds RNA of either gene leads to severe morphological defects in the ventral neuroectoderm [1].

### The formation of epithelial vesicles leads to a delay in neural differentiation

As revealed by DiI-labeling, secondary neural precursors within epithelial vesicles do not show any morphological features of differentiation. Obviously, the formation of epithelial vesicles after invagination leads to a delay in the differentiation of the corresponding neural precursors. Although the epithelial vesicles are formed at about the same time, they dissociate from each other subsequently. About half of them are still visible at the end of embryogenesis indicating that they provide neural precursors for larval stages.

In insects a distinct mechanism has evolved for generating larval neural precursors during embryonic life. After a phase of cell cycle arrest from late embryogenesis to first larval instar, 'embryonic' neuroblasts proliferate again. [9,10]. Both in *Drosophila melanogaster *and in *Manduca sexta*, the larval progeny of these neuroblasts accumulate in groups of cells that are separated by glial cell processes and do not finish their differentiation until the onset of metamorphosis [10,19]. It has been shown that the secreted glycoprotein anachronism (ana) regulates release of central brain neuroblasts from cell cycle arrest [20]. Ana is expressed in glial cells that ensheath central brain and optic lobe neuroblasts. In *ana *mutant larvae, neuroblasts proliferate earlier than in normal development which in turn leads to a premature differentation of neurons in certain brain regions. This heterochronic defect has an impact on the axonal pattern: the *ana *mutant phenotype ranges from subtle missrouting of fiber tracts to massive disorganization that affects the entire optic lobe [20]. These data show that factors regulating the differentiation state of neural precursors can have an important influence on the organization of neural networks.

The distinct morphology of the sheath cells in the spider neuromeres, i.e. their translucent cytoplasm, the absence of microvilli and the extension of cell processes that enwrap the neural precursors suggests that these are glial cells. Further analysis will show if these cells express genes that can influence the epithelial organization, i.e. the differentiation state of the secondary neural precursors, comparable to the glial cells of *Drosophila melanogaster*.

### Formation of epithelial vesicles – a basal mode of neurogenesis?

A recent study on neurogenesis in the onychophoran *Euperipatoides kanangrensis *shows that, rather than forming individual invagination groups, the whole medial regions of the hemi-segments invaginate into the embryo [21]. The invaginated cells remain attached to each other forming transitory epithelial vesicles. Although the phylogenetic position of Onychophora is still being debated, they are generally placed basally in the arthropodan clade [22-27]. Since onychophorans have retained many pleisiomorphic features, it can be assumed that they reflect a basal mode of CNS development [28-30]. This leads to the following model of changes in neural precursor formation during arthropod evolution: the basal mode of neurogenesis is the invagination of one large cluster of neural precursors from the central region of each hemi-neuromere. These clusters form transitory epithelial vesicles in the ventral neuromeres [31]. An advanced mode of neurogenesis is seen in chelicerates and myriapods: groups of cells that arise in several waves at stereotyped positions invaginate form the ventral neuroectoderm [1,3,4]. Interestingly, both chelicerate and myripod neurogenesis reflects some ancestral features. In the spider, epithelial vesicles are formed by secondary invaginating cell groups, while in myriapods the whole central regions of the hemi-neuromeres sink into the embryo after invagination of individual groups of neural precursors [3,32]. An even complexer mode of neurogenesis is seen in insects and crustaceans: individual neuroblasts are singled out from the ventral neuroectoderm that divide in sterotyped patterns to give rise to ganglion mother cells and finally neurons [33-42].

## Conclusions

To summarize, the model suggests that the invagination of large groups of neuroepithelial cells that form transient epithelial vesicles represents the basal mode of neurogenesis. Subsequently, more parameters have been introduced to the process of neurogenesis during arthropod evolution, i.e. sequential invagination/delamination of neural precursors and connection between neural precursor formation and cell proliferation. It can be assumed that these additional parameters have contributed to the diversity of neural precursor populations. This diversity might have been used as an evolutionary tool to develop neural networks that are adapted to the specialized behaviour and morphologies of the individual arthropod groups.

## Materials and Methods

### *Cupiennius salei *stocks

Fertilized females of the Central American wandering spider *Cupiennius salei *Keyserling (Chelicerata, Arachnida, Araneae, Ctenidae) were obtained from Ernst-August Seyfarth, Frankfurt, Germany. Embryos were collected as described before [1].

### Histology and stainings

Whole-mount in situ hybridisations were performed as described [1]. Phalloidin-rhodamine staining of spider embryos was performed as has been described for flies [43]. Anti-Phospho-Histone 3 immunocytochemestry has been performed as described [1].

### DiI-labeling

After chemically removing the chorion, embryos were fixed in 4 % formaldehyde in PBS and 1 vol heptane. The vitelline membrane was removed with needles and the embryos stained with phalloidin-FITC. Flat preparations of these embryos were attached to a coverslip with a double-sticky tape and covered with PBS. 1,1'-dioctadecyl 3,3,3',3'-tetramethyl indocarbocyanine perchlorate (DiI) was dissolved in ethanol and applied with glass needles. A small droplet of DiI was injected into single or several cells of an invagination group using a 63 × water-immersion lens and a FITC filter on a Zeiss fixed stage microscope and a micromanipulator.
